# Supplementary material for: Decreased CSTB, RAGE, and Axl Receptor Are Associated with Zika Infection in the Human Placenta
Source: Cells. 2022 Nov 16;11(22):3627. doi: 10.3390/cells11223627 (PMC9688057; doi:10.3390/cells11223627)
Supplement: Supplementary file 1 [file cells-11-03627-s001.zip › cells-2006303-supplementary.pdf]

Supplementary

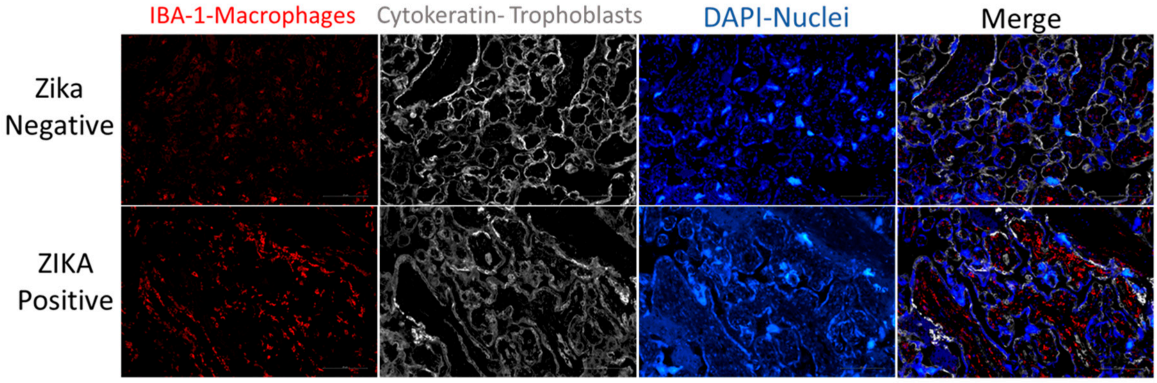

Supplementary Figure S1. Macrophage labeling of placental tissue in Zika negative and Zika positive placentas. Representative fluorescence immunohistochemistry of ZIKV uninfected placental tissue and ZIKV infected tissue. Protein labeling of Zika positive and Zika negative placentas expression in the placenta. The tissue is labeled with DAPI for nuclei (Blue), with anti-Iba1 for placental macrophages or Hofbauer cells (Red) and with anti-cytokeratin 8+18 (Pink) for trophoblast cells. Pictures were captured at a magnification of 20X.

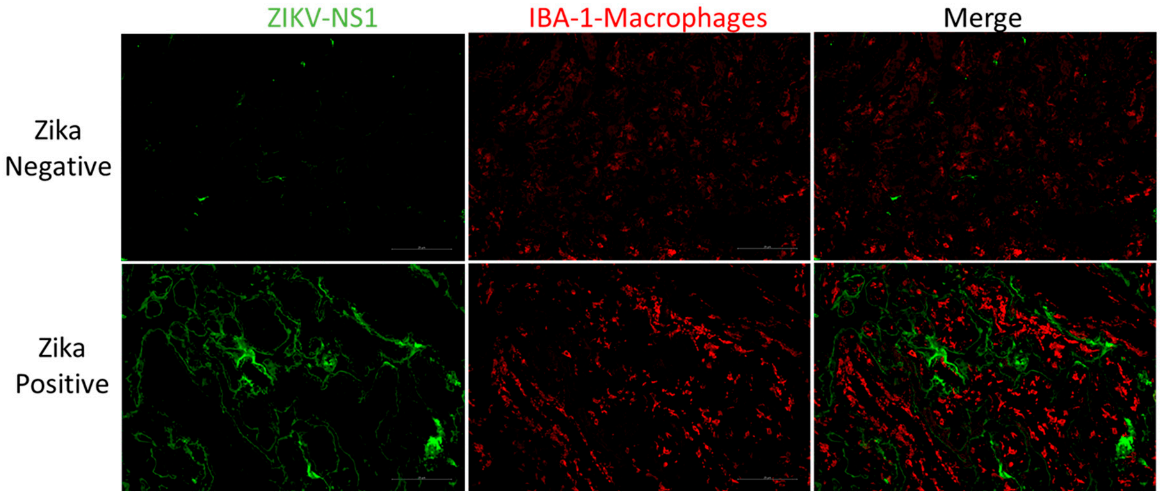

Supplementary Figure S2. Localization of Zika Non-Structural Protein 1 of placental tissue in Zika negative and Zika positive placentas. Representative fluorescence immunohistochemistry of ZIKV uninfected placental tissue and ZIKV infected tissue. Protein labeling of Zika positive and Zika negative placentas expression in the placenta. The tissue is labeled with DAPI for nuclei (Blue), with anti-Iba1 for placental macrophages or Hofbauer cells (Red) and anti-NS1 (Green). Pictures were captured at a magnification of 20X.

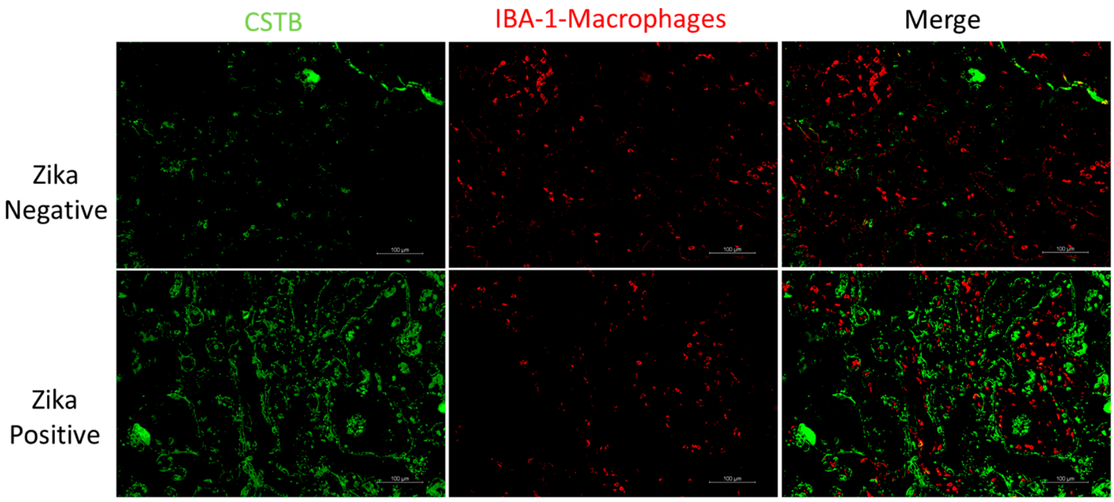

Supplementary Figure S3. Localization of Cystatin B in placental tissue of Zika negative and Zika positive placentas. Representative fluorescence immunohistochemistry of ZIKV uninfected placental tissue and ZIKV infected tissue. Protein labeling of Zika positive and Zika negative placentas expression in the placenta. The tissue is labeled with DAPI for nuclei (Blue), with anti-Iba1 for placental macrophages or Hofbauer cells (Red) and anti-NS1 (Green). Pictures were captured at a magnification of 20X.

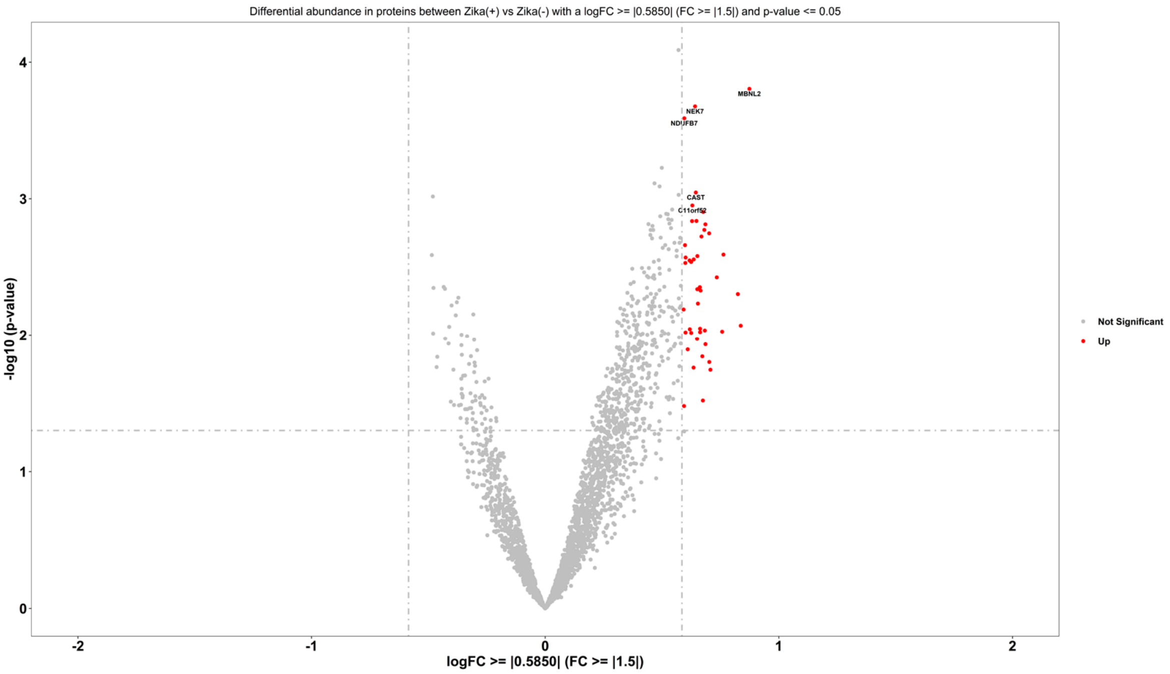

Supplementary Figure S4: Volcano plot for comparison between Zika(+) vs. Zika(-). The statistical analysis performed was a single channel analysis between cases vs. controls. The results from the statistical analysis were considered as proteins differentially abundant between groups based on FC ≥ |1.5| and p-value ≤ 0.05. More abundant (red dots) and no difference in abundance (grey dots).

Accession ID	Name	logFC	FC	pValue
O43148	mRNA cap guanine-N7 methyltransferase	0.6007	1.516	0.0095
O43865	S-adenosylhomocysteine hydrolase-like protein 1	0.5995	1.515	0.0030
O95361	Tripartite motif-containing protein 16	0.6284	1.546	0.0015
P02545	Prelamin-A/C	0.6350	1.553	0.0174
P04792	Heat shock protein beta-1	0.6356	1.554	0.0028
P07437	Tubulin beta chain	0.6247	1.542	0.0096
P08240	Signal recognition particle receptor subunit alpha	0.6245	1.542	0.0029
P09012	U1 small nuclear ribonucleoprotein A	0.6858	1.609	0.0117
P17568	NADH dehydrogenase [ubiquinone] 1 beta subcomplex subunit 7	0.5952	1.511	0.0003
P20337	Ras-related protein Rab-3B	0.5921	1.507	0.0065
P20810-6	Isoform 6 of Calpastatin	0.6444	1.563	0.0009
P23246	Splicing factor, proline- and glutamine-rich	0.7572	1.690	0.0094
P26196	Probable ATP-dependent RNA helicase DDX6	0.6649	1.585	0.0047
P29466	Caspase-1	0.6628	1.583	0.0089
P50443	Sulfate transporter	0.7015	1.626	0.0018
P52943	Cysteine-rich protein 2	0.7022	1.627	0.0158
P68104	Elongation factor 1-alpha 1	0.6808	1.603	0.0017
Q00839	Heterogeneous nuclear ribonucleoprotein U	0.6099	1.526	0.0127
Q07666	KH domain-containing, RNA-binding, signal transduction-associated protein 1	0.6831	1.606	0.0092
Q08379	Golgin subfamily A member 2	0.8245	1.771	0.0050
Q14157-1	Isoform 2 of Ubiquitin-associated protein 2-like	0.8367	1.786	0.0085
Q15365	Poly(rC)-binding protein 1	0.6508	1.570	0.0046
Q5VZF2	Muscleblind-like protein 2	0.8737	1.832	0.0002
Q6VY07	Phosphofurin acidic cluster sorting protein 1	0.6470	1.566	0.0015
Q6ZN17	Protein lin-28 homolog B	0.6746	1.596	0.0302
Q8N0X7	Spartin	0.6685	1.589	0.0019
Q8N370	Large neutral amino acids transporter small subunit 4	0.6533	1.573	0.0059
Q8N3F8	MICAL-like protein 1	0.5940	1.509	0.0331
Q8TDX7	Serine/threonine-protein kinase Nek7	0.6412	1.560	0.0002
Q8WU90	Zinc finger CCCH domain-containing protein 15	0.6189	1.536	0.0090
Q92625	Ankyrin repeat and SAM domain-containing protein 1A	0.6761	1.598	0.0012
Q92734	Protein TFG	0.7340	1.663	0.0038
Q96A22	Uncharacterized protein C11orf52	0.6296	1.547	0.0011
Q96CW1	AP-2 complex subunit mu	0.6176	1.534	0.0028
Q96PU8	Protein quaking	0.7628	1.697	0.0026
Q99700	Ataxin-2	0.6498	1.569	0.0107
Q9BQE3	Tubulin alpha-1C chain	0.6618	1.582	0.0044
Q9BZV2	Thiamine transporter 2	0.6721	1.593	0.0143
Q9H4A3	Serine/threonine-protein kinase WNK1	0.7070	1.632	0.0180
Q9P016	Thymocyte nuclear protein 1	0.6628	1.583	0.0095
Q9UBV8	Peflin	0.6855	1.608	0.0015
Q9UNH7	Sorting nexin-6	0.6007	1.516	0.0027
Q9Y2D5	A-kinase anchor protein 2	0.5981	1.514	0.0022
Q9Y5X1	Sorting nexin-9	0.6509	1.570	0.0026

Supplementary Table S1. Differentially abundant proteins were identified per group comparison with a Fold Change ≥ |1.5| and p-value ≤ 0.05.

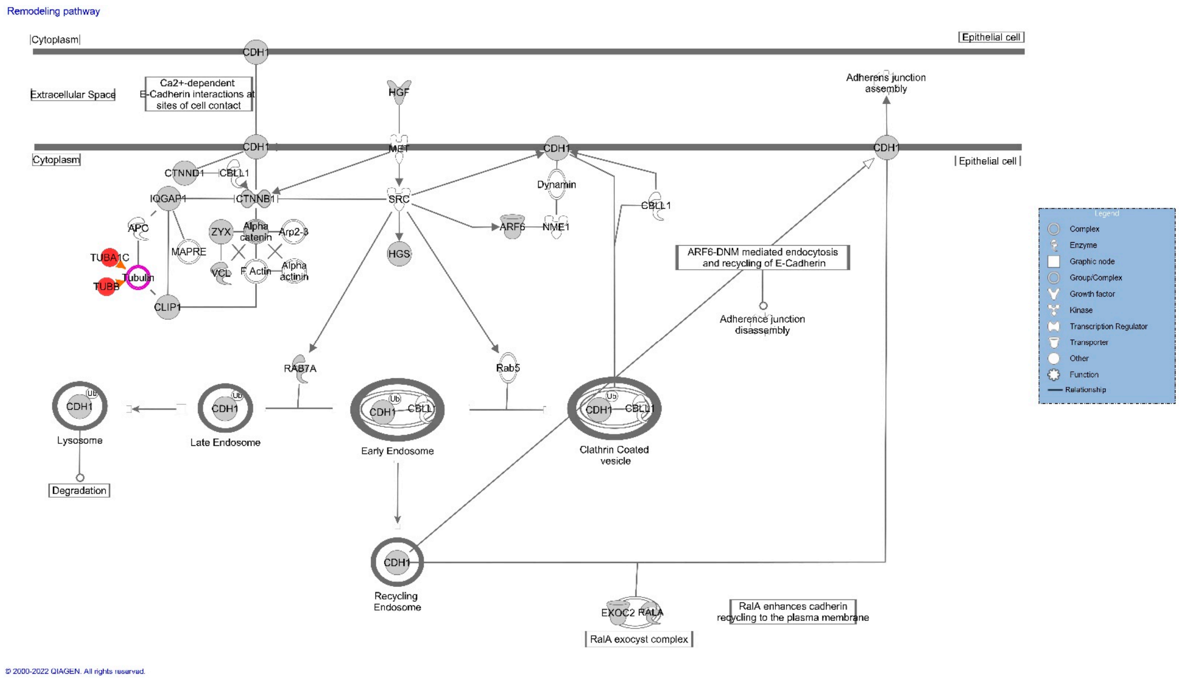

Supplementary Figure S5. Overexpression of Tubulin in Remodeling of Epithelial Adherent Junctions pathway. Prediction legend, the intensity of red means higher upregulation of the protein. These proteins are the tubulin alpha-1C chain (TUBA1C) and the tubulin beta chain (TUBB). The grey means that protein was identified, but there was no significant increase in expression. Data was analyzed using IPA (QIAGEN Inc., https://www.qiagenbioinformatics.com/products/ingenuitypathway-analysis, accessed 15/09/22).
